# Trends and factors associated with HIV testing among women aged 15–49 years in Belize: an analysis using the Multiple Indicator Cluster Survey 2006, 2011, and 2015–2016

**DOI:** 10.1186/s12905-023-02313-3

**Published:** 2023-04-11

**Authors:** Naudia L. Leonardo, Li-Yin Chien

**Affiliations:** 1 International Health Program, National Yang Ming Chiao Tung University, Yang-Ming Campus, Taipei, Taiwan; 2 Government of Belize, Ministry of Health and Wellness, Bliss Parade Belmopan, Belmopan, Belize; 3 Institute of Community Health Care, Collage of Nursing, National Yang Ming Chiao Tung University, Yang-Ming Campus, Taipei, 112304 Taiwan

**Keywords:** Human immunodeficiency virus, HIV testing, Belize, Multiple Indicator Cluster Survey, Reproductive age, Women

## Abstract

**Background:**

Belize has one of the highest human immunodeficiency virus (HIV)/acquired immunodeficiency syndrome prevalence rates in Central America, with women of reproductive age being particularly vulnerable to HIV. Therefore, this study examined the factors associated with HIV testing among women of reproductive age in Belize and trends in HIV testing in 2006, 2011, and 2015–2016.

**Methods:**

Cross-sectional data were analyzed using three Belize Multiple Indicator Cluster Surveys. The number of participants were 1,675, 4,096, and 4,699 women aged 15–49 years in 2006, 2011, and 2015–2016, respectively. We used variance-weighted least-squares regression to estimate annual changes. Multivariate logistic regression analysis was performed to evaluate the associated factors. Analyses were conducted using Stata version 15, and weights were applied for generalization to the population.

**Results:**

HIV testing rates increased from 47.7% in 2006 to 66.5% in 2015, with an average annual change of 0.082 (95% confidence interval: 0.07–0.09). Logistic regression models showed that women aged 15–24 years were less likely to have been tested for HIV compared to women aged 25–34 years. Women from the Mayan ethnic group were less likely to have been tested than those from other ethnic groups. Compared to women who spoke Spanish, those who spoke English/Creole were more likely to have been tested for HIV; additionally, those who spoke minority languages were less likely to have been tested. Being married and having given birth were associated with increased odds of HIV testing. Living in rural areas and households with the poorest wealth indices were associated with decreased odds of being tested for HIV. Women with good HIV knowledge and accepting attitudes towards people living with HIV were more likely to be tested.

**Conclusions:**

From 2006 to 2015, HIV testing in women of reproductive age showed an increasing trend in Belize. We recommend interventions to expand HIV testing for women of reproductive age in Belize, particularly those aged 15–24 years, speaking minority languages, living in rural areas, and having a low socioeconomic status.

## Background

Human immunodeficiency virus (HIV) is a significant contributor to the global public health problem [1]. In 2017, the United Nations Agency for International Development (UNAIDS) reported that Belize, a middle-income country in Latin America and the Caribbean, had the highest HIV prevalence rate (1.9%) in adults aged 15–49 years in Latin America and the fourth highest in the Caribbean [2]. In Belize, there were approximately 200–300 new cases of HIV diagnosed annually, with an incidence rate of 0.93 in 2017 [3]. 

Knowledge of one’s HIV status is crucial to improve quality of life, prolong life expectancy, and make behavioral changes. Hence, HIV testing is an integral part of HIV prevention and transmission [4]. Early HIV diagnosis can improve access to early treatment and limit further disease transmission [5].

New HIV infections in women aged 15–24 years worldwide reduced by 25% between 2010 and 2018. However, this group still accounted for 48% of all newly diagnosed HIV infections among adults in 2019 [6, 7]. **S**ome countries have shown a different trend in HIV, such as Belize, which was the only country in the Caribbean with a 7% increase in new HIV infections between 2010 and 2018 [8].

In 2003, Voluntary Counselling and Treatment (VCT) services and free HIV treatment programs providing antiretroviral drugs to all patients were introduced to the Belizean health system. In the same year, the Belize Ministry of Health and Wellness introduced the Prevention of Mother-to-Child Transmission of HIV (PMTCT) program, in which pregnant women are tested for HIV as part of their routine antenatal screening. In 2006, the National Health Insurance scheme was introduced, and primary healthcare services, including HIV testing in rural and remote areas, had improved. A formal HIV/acquired immunodeficiency syndrome (AIDS) policy and strategic direction, i.e., the HIV/AIDS Policy of the Public Service of Belize, was published in 2007 [9]. The policy statement and action plan included: (1) no mandatory HIV testing is to be carried out while voluntary testing for HIV/AIDS is encouraged with appropriate pre- and post-test counseling services made available and accessible; (2) information about the HIV status should be treated confidentially; (3) stigma and discrimination towards people living with HIV (PLHIV) and AIDS should be reduced; and (4) information and awareness-raising campaigns, educational programs, linkage to health promotion programs, and community outreach programs on HIV/AIDS should be made available. This strategic direction was maintained throughout the data period (2005–2016) [9].

Many factors influence the uptake of HIV testing among women, such as age; residence; marital status; education; HIV knowledge; pregnancy history; and physical, social, economic, and political factors. Improving of HIV awareness and HIV education has positively impacted women of all ages [10–12]. Several studies have outlined barriers to HIV testing, such as the belief that one was not at risk of contracting HIV, proximity to home, inconvenience of testing, multiple partnerships, and negative attitudes towards HIV [13–15]. Those who had not tested for HIV showed higher stigma to AIDS and shame, guilt, and social disapproval towards PLHIV [16]. Heterosexual unprotected intercourse is the primary route of HIV transmission among Belizean women, accounting for one-third of all new HIV infections. Other extrinsic factors include the inconsistent condom use in the presence of multiple partners, early sexual initiation, and gender‐based violence [3]. Men who have sex with men account for the largest share of the HIV epidemic, with approximately 1,500 men living with HIV and two-thirds of all new HIV infections [3].

Understanding the factors that contribute to the low rate of HIV testing in women of reproductive age is essential. These factors, if addressed, can contribute to an increase in HIV testing uptake in women of reproductive age [10]. Testing for HIV is crucial in curbing the epidemic and reaching the UNAIDS goal of 95% of PLHIV being tested and made aware of their HIV status. This study examined factors associated with HIV testing in women of reproductive age in Belize and trends in HIV testing in 2006, 2011, and 2015–2016.

## Methods

### Study design, study period, and data source

Data for this cross-sectional study were retrieved from three waves of Multiple Indicator Cluster Surveys (MICS) conducted in Belize in 2006, 2011, and 2015–2016. The surveys were conducted by the Statistical Institute of Belize in collaboration with the United Nations Children’s Fund (UNICEF). The MICS database is the largest source of global statistical data on children aged < 5 years and women aged 15–49 years [17].

### Sampling and study participants

Data were collected using multistage stratified cluster sampling to select nationally representative households. In the 2006 survey, six administrative zones were identified; in the 2011 and 2015–2016 surveys, seven zones were identified. Clusters of primary sampling units were selected from each stratum using a systematic probability proportional to size, based on the number of households in the enumeration districts in the first stage of sampling. The number of households in each district was determined using a national census sample frame. The second stage involved the systematic selection of households within each enumeration district. All variables for this study were from women's datasets for 2006 (*N* = 1,675), 2011 (*N* = 4,096), and 2015–2016 (*N* = 4,699). The final analytical dataset comprised 10,470 respondents with complete data on the variables of interest.

### Outcome variable

HIV testing results were used as the outcome variable. To assess HIV testing, respondents were asked if they had ever been tested for HIV. A binary outcome variable was created for HIV testing, coded “0” if the respondent had never been tested and “1” if the respondent had been tested for HIV.

### Explanatory variables

The explanatory variables for this study were based on previous studies and the availability of such variables in the dataset. Age was categorized into three groups: 15–24 years, 25–35 years, and 35–49 years. Ethnicity was categorized as Creole, Mestizo, Garifuna, Maya, or others. Religion was divided into Roman Catholics, Protestants, Others, and None. Marital status was categorized as never married, currently married/in union, and formerly married/formerly in union, and education was dichotomized into none/primary and secondary/higher. “Ever given birth” was a yes/no variable. The household wealth index was used as a proxy variable for economic status. The methods used to calculate wealth index was adopted from the USAID Demographic Health Survey [18]. The household wealth index was ranked into quintiles from poorest, second poorest, middle, fourth, and richest [18].

#### HIV knowledge and attitude

The HIV/AIDS knowledge and attitude variables were constructed using questions from the HIV/AIDS module of MICS. Knowledge was measured by dichotomizing each item into a value of “1” (correct) or “0” (incorrect or unknown). To calculate the HIV knowledge score, correct answers were added with a range of 0–7 points. A composite score of ≥ 5 was classified as having good knowledge of HIV and ≤ 4 was classified as having poor knowledge of HIV. Internal consistency was checked using Cronbach’s alpha, which was 0.83 for the HIV knowledge scale. The HIV attitude scale was measured by dichotomizing each item into values of “1” (positive) and “0” (negative or unknown). Positive answers were added to calculate an HIV attitude score ranging from 0–4. Scores ≥ 3 and ≤ 2 indicated an accepting and rejecting attitude towards PLHIV, respectively. Internal consistency was checked using Cronbach’s alpha, which was 0.61 for the HIV attitude scale. The cutoff scores for the establishment of good/poor knowledge and accepting/rejecting attitude variables were set by the MICS Team and used in previous studies [19, 20].

### Statistical analysis

Statistical analyses were conducted using Stata Statistical Analysis Version 15.1. Descriptive statistics in the form of percentages were calculated for characteristics of the study population. Weighted percentages were used to calculate the descriptive analysis. Variance-weighted least-squares regression was used to estimate the annual changes in HIV testing during the data period. Multivariate logistic regression models were used to analyze the effects of HIV knowledge, attitudes, and sociodemographics on HIV testing. All multivariate models in the study were controlled for the covariates, and results were presented as adjusted odds ratios (aOR) with their corresponding 95% confidence intervals (CI); statistical significance was set at *p* < 0.05. To ensure nationally representative estimations and generalizability to the population, the survey sampling weights provided in the MICS dataset were utilized in the regression models for the yearly analysis. The variance inflation factor (VIF) was calculated for all independent variables included in the regression analysis, and all VIF values were below 2.

### Ethical considerations

All the participants signed an informed consent form for the MICS survey. For those younger than 18 years, assent from the underage participants and informed consent signed by a parent or legal guardian were obtained. The study was conducted in accordance with the UNICEF procedure for ethical standards in research, evaluation, data collection and analysis [21]. Anonymous data were retrieved. The National Yang Ming Chiao Tung University Institutional Review Board approved using of the MICS survey data and ensured that this study abided by all ethical considerations (No. YM110106E).

## Results

### Trends in HIV testing status

**Fig. 1:**
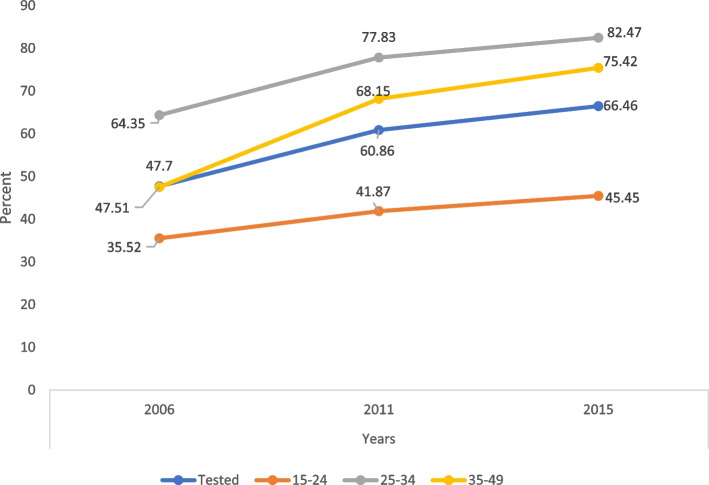
Trends in ever-tested rates in women by age groups in Belize 2006, 2011, and 2015

HIV testing rates increased from 47.70% in 2006 to 60.86% in 2011 and 66.46% in 2015–2016. Testing in the age group 15–24 years increased from 35.52% in 2006 to 45.45% in 2015–2016. Testing in the age group 35–49 years increased from 47.51% in 2006 to 75.42% in 2015–2016. The age group 25–34 years had the highest testing rate across the time period, which increased from 64.35% in 2006 to 82.47% in 2015–2016 (Fig. 1).

The variance-weighted least-squares regression showed that the average annual change in testing was 0.082 (95% CI: 0.07–0.09) during 2006–2015. For age groups 15–24 years, 25–34 years, and 35–49 years, the average annual change was 0.047 (95% CI: 0.03–0.07), 0.075 (95% CI: 0.05–0.10), and 0.123 (95% CI: 0.10–0.15), respectively.

### Characteristics of the study population

As shown in Table 1, approximately half of participants were from the Mestizo ethnic group (47.79% in 2006, 49.96% in 2011, and 51.25% in 2015) and another 24% were from the Creole ethnic group (24.61% in 2006, 24.04% in 2011, and 24.81% in 2011). About 38% were aged 15–24 years (37.71 in 2006, 38.18% in 2011, and 38.01% in 2015). About 45% spoke Spanish as their first language (43.64% in year 2006, 45.42% in 2011, and 44.24% in 2016) and 39% spoke English/Creole as their first language (38.31% in 2006, 39.16% in 2011, and 39.52% in 2015). Approximately 60% were currently married (58.28% in 2011, 62.46% in 2015). About two-thirds had given birth (68.65% in 2006, 66.61% in 2011, and 65.56% in 2015). Approximately half of the respondents were from rural areas (47.93% in 2006, 52.99% in 2011, and 54.84% in 2015). The highest proportion of residency was in Belize City (30.3%) in 2006, Cayo (22.78%) in 2011, and Stann Creek (22.58%) in 2016. More women had secondary or higher levels of education in 2011 and 2015 (57.12% in 2011, 57.69% in 2015) than in 2006 (45.73%). Approximately 16.0% of the participants were from households in the poorest quintile (16.87% in 2006, 15.73% in 2011, and 16.91% in 2015). Approximately 82% of women demonstrated good knowledge about HIV (80.56% in 2006, 83.67% in 2011, and 82.94% in 2015). More women had an accepting attitude towards PLHIV in 2015 (58.36%) and 2011 (56.89%) than in 2006 (45.71%).

**Table 1:** Characteristics of women surveyed in the Belize MICS in 2006, 2011, and 2015–2016

Variables (Total)	2006	2011	2015–2016
	*n* = 1,675 (%)	*n* = 4,096 (%)	*n* = 4,699 (%)
**Age-group (Years)**			
15–24 years	37.71	38.18	38.01
25–34 years	28.59	29.17	30.14
35 +	33.70	32.65	31.85
**Ethnicity**			
Creole	24.61	24.04	24.81
Mestizo	47.79	49.96	51.25
Garifuna	6.69	6.19	5.36
Maya	11.87	9.94	10.72
Other	9.04	9.88	7.86
**Language**			
English/Creole	38.31	39.16	39.52
Spanish	43.64	45.42	44.24
Other	18.05	15.42	16.24
**Marital Status**			
Currently married	N/A	58.28	62.46
Formerly married	N/A	11.94	10.48
Never married	N/A	29.78	27.06
**Ever given birth**			
No	31.35	33.39	34.44
Yes	68.65	66.61	65.56
**Religion**			
Roman Catholic	51.24	40.93	37.79
Protestant/Other	40.92	46.26	45.25
None	7.84	12.81	16.95
**Area of Residence**			
Urban	52.07	47.01	45.16
Rural	47.93	52.99	54.84
**District of Residency**			
Corozal	15.05	13.04	12.48
Orange Walk	14.64	15.09	15.68
Belize City	30.30	16.77	17.12
Belize Southside	N/A	13.98	9.00
Cayo	21.18	22.78	13.22
Stann Creek	10.61	9.87	22.58
Toledo	8.22	8.47	9.92
**Women’s Education**			
None/Primary	54.27	42.88	42.31
Secondary/Higher	45.73	57.12	57.69
**Wealth Index Quintiles**			
Poorest	16.87	15.73	16.91
Second	19.04	19.91	20.13
Middle	20.61	21.40	21.18
Fourth	21.34	21.05	21.75
Richest	22.15	21.91	20.03
**HIV Knowledge**			
Poor knowledge	19.44	16.33	17.06
Good Knowledge	80.56	83.67	82.94
**Attitude towards HIV**			
Rejecting attitudes	54.29	43.11	41.64
Accepting attitudes	45.71	56.89	58.36

*MICS* Multiple Indicator Cluster Surveys, *HIV* Human immunodeficiency virus

### Responses to the HIV knowledge and attitudes questions

The mean knowledge score was 5.52 (standard deviation [SD] = 1.26, range = 0–7), and the mean HIV attitude score was 2.38 (SD = 1.29, range = 0–4). The proportion of respondents who had heard of AIDS decreased from 96.42% in 2006 to 89.79% in 2015. The transmission of HIV from mosquito bites had the lowest correct response with 68.24% in 2006, decreasing to 65.12% by 2015. Women's attitudes towards caring for a family member with the AIDS in one's own home had the highest positive response (77.31% in 2006, 78.27% in 2011, and 76.61% in 2015; Table 2).

**Table 2:** HIV knowledge and attitude responses in the Belize MICS in 2006, 2011, and 2015–2016

Questions	% of correct/accepting answers		
	2006	2011	2015–2016
	*n* = 1,675 (%)	*n* = 4,096 (%)	*n* = 4,699 (%)
**HIV Knowledge**			
Have you ever heard of AIDS?	96.42%	92.8%	89.79%
Can people reduce their risk of contracting HIV by having an uninfected sexual partner and no other sexual partners?	73.07%	76.05%	79.08%
Can people reduce their risk of contracting HIV by utilizing a condom on every sexual contact?	70.75%	71.22%	72.87%
Can a healthy-looking person have the AIDS virus?	84.60%	83.40%	82.17%
A mosquito bite cannot transmit HIV?	68.24%	68.14%	65.12%
Can people not get HIV through witchcraft or other supernatural means?	84.54%	84.16%	80.76%
Can HIV not be transmitted by sharing food with someone with AIDS?	76.18%	78.22%	76.27%
**HIV Attitude**			
Are you willing to care for a family member with the AIDS virus in own home?	77.31%	78.27%	76.61%
Would you buy fresh vegetables from a shopkeeper or vendor who has the AIDS virus	46.27%	53.30%	52.63%
Do you believe that a female teacher infected with the AIDS virus who is not sick should continue teaching?	54.87%	63.45%	65.80%
Would you not want to keep it secret that a family member got infected with the AIDS virus?	42.39%	43.19%	48.84%

*HIV* Human immunodeficiency virus, *MICS* Multiple Indicator Cluster Surveys, *AIDS* Acquired immunodeficiency syndrome

### Logistic regression between HIV testing and respondent characteristics

As shown in Table 3, in 2006, women who had given birth (aOR = 12.36, 95% CI: 8.43–18.13) were more likely to have tested for HIV than those who had not. Women whose first language was “other” (aOR = 0.48, 95% CI: 0.25–0.91) were less likely to have undergone HIV testing than those whose first language was “Spanish,” whereas there were no significant differences among women with first language being English/Creole/Spanish with regards to likeliness of HIV testing. Living in rural areas was associated with lower odds of HIV testing (aOR = 0.63, 95% CI: 0.48–0.81). Good knowledge was associated with higher odds of HIV testing compared to poor knowledge (aOR = 1.59, 95% CI: 1.15–2.21). Age, ethnicity, religion, district, education, wealth index, and attitudes were not significantly associated with the odds of HIV testing.

**Table 3:** Multiple logistic regression analysis of factors associated with being tested for HIV in women aged 15–49 years in Belize, by the survey year 2006, 2011, and 2015/2016

	HIV Testing in lifetime (Years)		
	2006	2011	2015–2016
	AOR (95% CI)	AOR (95% CI)	AOR (95% CI)
**Age groups (ref. 15–24 years)**			
25–34 years	1.00 (0.66–1.50)	1.61(1.25–2.11) ***	2.87 (2.18–3.79)***
35–49 years	0.37 (0.78–1.33)	0.52 (040–0.69)***	1.24 (0.91–1.68)
**Ethnicity (ref. Maya**)			
Creole	0.83 (0.50–1.57)	1.68 (0.98–2.90)	2.09 (1.08–4.04)**
Mestizo	1.15(0.63–2.11)	1.73 (1.01–2.95)*	1.23 (0.70–2.17)
Garifuna	1.34 (0.72–2.51)	3.09 (1.73–5.53)***	2.73 (1.40–5.29)**
Other	1.00 (0.56–1.79)	1.03 (0.60–1.77)	0.79 (0.45–1.34)
**Marital status (ref. Never married)**			
Currently married	N/A	2.95 (2.24–3.89)***	3.53 (2.65–4.72)***
Formerly married	N/A	3.13 (2.24–4.39)***	4.01 (2.80–6.22)***
**Ever give birth (ref. Never)**	12.36 (8.43–18.13)	8.72 (6.90–11.03)***	8.39 (6.09–11.57)***
**Language (ref. Spanish)**			
English/Creole	1.49 (0.97–2.28)	1.61 (1.16–2.24)**	0.95 (0.67–1.40)
Other	0.48 (0.25–0.91)*	0.75 (0.47–1.21)	0.35 (0.20–0.61)***
**Religion (ref. None)**			
Roman Catholic	0.99 (0.74–1.34)	0.99 (0.74–1.33)	0.93 (0.67–1.30)
Protestant/Other	0.98 (0.64–1.51)	1.10 (0.84–1.46)	0.75 (0.54–1.05)
**Area of residence (ref. Urban)**			
Rural	0.63 (0.48–0.81)***	0.90(0.72–1.13)	0.67 (0.51–0.87)**
**District of residency (ref. Toledo)**			
Corozal	0.83 (0.46–1.52)	1.92 (1.20–3.07)**	1.07 (0.70–1.66)
Orange Walk	0.79 (0.43–1.40)	2.22 (1.43–3.43)***	0.84 (0.55–1.29)
Belize City	1.44 (0.67–1.95)	2.00 (1.25–3.19)**	1.03 (0.65–1.61)
Belize Southside	N/A	2.69 (1.70–4.25)***	0.59 (0.39–0.87)*
Cayo	0.79 (0.45–1.40)	2.09 (1.36–3.19)**	0.78 (0.44–1.36)
Stann Creek	1.63 (0.93–2.88)	2.55 (1.69–3.84)***	0.49 (0.31–0.76)**
**Education (ref. None/Primary)**			
Secondary/Higher	1.30 (0.92–1.82)	1.06 (0.93–1.45)	1.18 (0.90–1.53)
**Wealth Index Quintiles (ref. Poorest)**			
Second	1.34 (0.88–2.04)	1.92 (1.27–2.25)***	1.52 (1.06–2.17)**
Middle	1.21 (0.75–1.93)	1.16 (0.87–1.55)	1.31 (0.88–1.93)
Fourth	1.41 (0.85–2.33)	1.51 (1.09–2.09)*	1.12 (0.77–1.62)
Richest	1.07 (0.65–1.77)	1.49 (1.06–2.09)*	1.30 (0.87–1.93)
**HIV knowledge (ref. Poor knowledge)**			
Good knowledge	1.59 (1.15–2.21)**	2.89 (2.21–3.78)***	6.89 (5.06–9.38)***
**Attitude to PLHIV (ref. Rejecting attitude)**			
Accepting attitude	1.06 (0.81–1.40)	1.32 (1.07–1.63)**	1.92 (1.55–2.42)***

2006 Dataset (*n* = 1,675), 2011 Dataset (*n* = 4,096), 2015/2016 Dataset (*n* = 4,699)

*aOR* Adjusted odds ratio, *CI* Confidence interval, *HIV* Human immunodeficiency virus, *PLHIV* People living with HIV

*P*-value *p < 0.05, **p < 0.01, ***p < 0.001

In 2011, women aged 25–34 years (aOR = 1.61, 95% CI: 1.25–2.11) were more likely to test for HIV, whereas women aged 35–49 (aOR = 0.52, 95% CI: 0.40–0.69) were less likely to test for HIV than those aged 15–24. Women of Mestizo (aOR = 1.73, 95% CI: 1.01–2.95) and Garifuna (aOR = 3.09, 95% CI: 1.73–5.53) ethnicities were more likely to have undergone HIV testing than those of Mayan ethnicity, whereas women of Creole and other ethnicities did not differ from those of Mayan group. Women who were currently married (aOR = 2.95, 95% CI: 2.24–3.89) and formerly married (aOR = 3.13, 95% CI: 2.24–4.39) had higher odds of testing for HIV than those who had never married. Women who had given birth (aOR = 8.72, 95% CI: 6.90–11.03) were more likely to have undergone HIV testing than those who had not. Women whose first language was English/Creole (aOR = 1.61, 95% CI: 1.16–2.24) were more likely to have undergone HIV testing than those whose first language was Spanish, whereas there were no significant differences between women whose first language was “other” and Spanish. In the Toledo region, those living in Corozal, Orange Walk, Belize City, Belize Southside, Cayo, and Stann Creek were more likely to undergo HIV testing. Women with higher wealth index compared to those with poorer wealth index were associated with increased odds for HIV testing; the difference was statistically significant for second, forth, and richest wealth index but was not significant for middle wealth index. Good knowledge was associated with higher odds of HIV testing than poor knowledge (aOR = 2.89, 95% CI: 2.21–3.78). An accepting attitude towards PLHIV was associated with a higher odds of HIV testing than rejecting attitude (aOR = 1.32, 95% CI: 1.07–1.63). Religion, living in a rural area, and education were not significantly associated with HIV testing in this model.

During 2015–2016, women aged 25–34 years (aOR = 2.87, 95% CI: 2.18–3.79) were more likely to have undergone HIV testing, whereas women aged 35–49 years did not significantly differ from those aged 15–24 years in regards to likeliness of HIV testing. Creole (aOR = 2.09, 95% CI: 1.08–4.04) and Garifuna (aOR = 2.73, 95% CI: 1.40–5.29) ethnicities were more likely to have undergone HIV testing than the Mayan group, whereas Mestizo and other ethnicities did not differ significantly from Mayan ethnicity pertaining to the likeliness of HIV testing. Women who were currently married (aOR = 3.25, 95% CI: 2.65–4.72) and formerly married (aOR = 4.01, 95% CI: 2.80–6.22) were more likely to undergo HIV testing than those who had never married. Women who had given birth (aOR = 8.39, 95% CI: 6.09–11.57) were more likely to have tested for HIV than those who had not. Women whose first language was “other” (aOR = 0.35, 95% CI: 0.20–0.61) were less likely to have tested for HIV than those whose first language was Spanish, whereas there were no significant differences between women whose first language was English, Creole, or Spanish with regards to likeliness of HIV testing. Living in a rural area was associated with lower odds of HIV testing (aOR = 0.67, 95% CI: 0.51–0.87). With reference to the Toledo region, those living in Belize Southside and Stann Creek were more likely to undergo HIV testing. Compared to the poorest wealth, the second wealth index were associated with significantly increased odds for HIV testing (aOR = 1.52, 95% CI: 1.06–2.17). While the middle, fourth, and richest wealth indices had higher odds for HIV testing than the poorest, the aORs were not statistically significant. Good knowledge was associated with higher odds of HIV testing than poor knowledge (aOR = 6.89, 95% CI: 5.06–9.38). An accepting attitude was associated with higher odds of HIV testing than a rejecting attitude (aOR = 1.92, 95% CI: 1.55–2.42). Religion and education were not significantly associated with HIV testing.

In all three models, giving birth and good HIV knowledge were associated with an increased odds of HIV testing. Living in rural areas was associated with decreased odds of HIV testing, although statistically significant differences were observed in 2006 and 2015 but not in 2011. Those of Mayan ethnicity were less likely to undergo HIV testing in 2011 and 2016. Women who were currently and formerly married were associated with increased odds of HIV testing compared to those who had never married. Women speaking “other” language were less likely to undergo HIV testing than those whose first language was “Spanish.” These differences were statistically significant for 2006 and 2015 but not for 2011. Differences in district areas in regards to HIV testing between 2011 and 2016 were observed, and the differences across districts in 2011 differed from those in 2016. Those with the poorest wealth index were less likely to have undergone HIV testing in 2011 and 2016 compared to those with richest wealth indices. Accepting attitudes were associated with higher odds of HIV testing than rejecting attitudes, although statistically significant differences were found in 2011 and 2015 but not in 2006.

## Discussion

To our knowledge, this is the first study to analyze the factors associated with both HIV testing and HIV testing trends among women of reproductive age in Belize. Our analysis of three MICS Surveys (2006, 2011, and 2015–2016) in Belize revealed significant increases in HIV testing among women of reproductive age, which could be attributed to several policy initiatives. In 2006, USAID developed indicators to monitor the quality of care provided to PLHIV and those living with AIDS. Bi-annual quality audits were conducted at all public health facilities including hospitals and clinics to ensure that counseling was conducted pre- and post-HIV testing and to ensure that women were enrolled in the PMTCT program [9]. In 2007, the HIV/AIDS Policy of the Public Service of Belize was implemented. This policy focused on providing enabling environment for all and providing quality and efficient public services to contribute to the development of Belize [9]. The Ministry of Health, to increase testing for HIV support and fund health fairs, provided health education sessions and VCT for HIV and provider-initiated testing and counseling, which are now available at all healthcare institutions in Belize. This made HIV testing widely available and accessible to the broader population accessing health services [2, 3, 9]. Close monitoring and audits, service improvement, and public awareness and community programs contributed to the increase in the uptake in HIV testing [2, 4, 9].

Belize experienced an average annual change of 0.08 in HIV testing between 2006 and 2015. This trend was pervasive across all age groups. The prevalence of HIV testing was the highest for those aged 25–34 years and the lowest for those aged 15–24 years, with ages 35–49 years having an intermediate prevalence throughout the study period. The average annual change was highest for age group 35–49 years (0.12), followed by age group 25–34 years (0.08) and the lowest for age group 15–24 years (0.05). These results suggest that further promotion of HIV testing among women aged 15–24 years is required.

This study outlined the sociodemographic factors, HIV knowledge, and attitudes associated with HIV testing among women aged 15–49 years in Belize. We built predictive models for 2006, 2011, and 2015. Although some variables were significant in only one or two waves, the direction of the association was usually consistent across time. We believe that the associated variables did not differ across time periods except for the districts of residency. Toledo had lower HIV testing rates than all other districts in 2011; however, it had higher HIV testing rates than Belize Southside and Stann Creek in 2015. Further studies are needed to examine district-based differences in HIV testing rates.

Factors associated with testing included age, ethnic group, marital status, childbirth, language, living in rural areas, wealth index, knowledge of HIV, and attitudes towards PLHIV. In this study, participants aged 15–24 years were less likely to undergo HIV testing than those aged 25–34. Similar findings were noted in other studies from the United States of America [22], the Dominican Republic [13], Kenya [15], and Ethiopia [23]. In Belize, the low uptake of HIV testing in adolescents may be associated with the higher legal age of consent for HIV testing, which is 18 years [24].

Ethnicity was significantly associated with HIV testing. Women of Garifuna (2011, 2015), Creole (2015), and Mestizo (2011) ethnicities were more likely to be tested for HIV than those from Mayan ethnic group were. The Mayans are the only indigenous culture comprising the majority of the population in Central America [25]. A study in Guatemala concluded that although young Mayan adults knew where they could be tested for HIV, they did not access the service because of privacy issues and fear of a positive diagnosis [26].

Parity and marital status were positively associated with HIV testing. The respondents who had given birth were more likely to have been tested for HIV, which could be correlated with the PMTCT program. Those who are married or in a partnership may sense the personal danger of contracting HIV in previous or present relationships, prompting them to seek HIV testing [27].

Compared to those whose first language was Spanish, speaking English/Creole was associated with increased odds of undergoing HIV testing (2011), whereas speaking a language other than English/Creole/Spanish was associated with decreased odds of undergoing HIV testing. This finding can be explained by the official language of Belize being English [25]. Limited access to care, low educational level, inadequate English proficiency, and language barrier in the access and uptake of HIV testing can be multifaceted for both the patient and the healthcare provider. Numerous primary healthcare facilities in Belize have employed physicians from the Cuban Brigade, whose primary language is Spanish, thereby creating a barrier for those who speak Mayan or other languages [27, 28].

Despite the fact that HIV testing in Belize is free, women from the poorest household wealth index quintile had decreased odds of undergoing HIV testing (2011, 2015) because of the unavailability of funds to travel to health facilities [11, 27, 28]. Rurality was associated with a lower likelihood to undergo HIV testing than urban areas. Healthcare in Belize tends to be centralized in urban areas; therefore, people from rural areas must travel long distances to access healthcare. Similar findings were noted for Ethiopia [29], Ghana [30], and Tajikistan [10]. The Government of Belize has initiated the building of health posts and satellite clinics in the rural areas; however, owing to poor road conditions and financial constraints, accessing health services in rural area still poses a significant burden [27].

Good knowledge and accepting attitudes towards PLHIV were associated with increased uptake of HIV testing, suggesting that poor knowledge of HIV/AIDS and rejecting attitudes towards PLHIV constitute significant barriers to undergoing an HIV test. Similar studies have reported that individuals with good HIV knowledge were associated with an increased likelihood of undergoing an HIV test, whereas those with a negative attitude towards PLHIV were less likely to get tested for HIV [11, 22]. In Belize, outreach training is conducted to educate the public about HIV through community health workers and health educators [27].

### Study strengths and limitations

This study had several strengths. A dataset from a nationally representative survey sample was used. Furthermore, three waves of MICS datasets were utilized, resulting in a large survey sample size. MICS are widely perceived to be of optimal quality, since they are based on internationally tested questionnaires, sampling methodologies, and high response rates.

This study had several limitations. This was a cross-sectional design study, which does not allow inferring causality between associated factors and HIV testing. In addition, the responses were self-reported, with the risk of social desirability bias. As the models were built separately for different years, the aORs from the different models were not directly comparable.

## Conclusions

From 2006 to 2015, HIV testing in women aged 15–49 years showed an increasing trend in Belize. Interventions to expand HIV testing, such as changing the age of consent for HIV testing from 18 to 15 years and improving knowledge of HIV and attitudes towards PLHIV, should be implicated. We recommend interventions to expand HIV testing for women of reproductive age in Belize, particularly those aged 15–24 years, speaking minority languages, residing in rural areas, and those with a low socioeconomic status. HIV testing services should be made more accessible, especially for people living in rural areas who are economically challenged.

## Data Availability

Data is available upon reasonable request from the corresponding author.

## Abbreviations

HIV: Human Immunodeficiency Virus
AIDS: Acquired immunodeficiency syndrome
MICS: Multiple Indicator Cluster Survey
UNAIDS: United Nations Agency for International Development
UNICEF: United Nations Children's Fund
PLHIV: People living with HIV
aOR: Adjusted odds ratio
CI: Confidence interval
VCT: Voluntary Counselling and Testing
PMTCT: Mother-to-child transmission
